# FADS2 Polymorphisms Modify the Effect of Breastfeeding on Child IQ

**DOI:** 10.1371/journal.pone.0011570

**Published:** 2010-07-13

**Authors:** Colin D. Steer, George Davey Smith, Pauline M. Emmett, Joseph R. Hibbeln, Jean Golding

**Affiliations:** 1 Centre for Child and Adolescent Health, Department of Community Based Medicine, University of Bristol, Bristol, United Kingdom; 2 MRC Centre for Causal Analyses in Translational Epidemiology, University of Bristol, Bristol, United Kingdom; 3 National Institutes of Health, National Institute on Alcohol Abuse and Alcoholism, Bethesda, Maryland, United States of America; Instituto Gulbenkian de Ciência, Portugal

## Abstract

**Background:**

Breastfeeding is important for child cognitive development. A study by Caspi et al has suggested that rs174575 within the FADS2 gene moderates this effect so that children homozygous in the minor allele (GG genotype) have similar IQs irrespective of feeding method.

**Methods and Principal Findings:**

In our study of 5934 children aged 8 years, no genetic main effect with IQ was found for rs174575. However, an interaction with this polymorphism was observed such that breastfed GG children performed better than their formula fed counterparts by an additional 5.8 points [1.4, 10.1] (interaction p = 0.0091). Interaction results were attenuated by about 10% after adjustment for 7 factors. This study also investigated rs1535, another FADS2 polymorphism in linkage disequilibrium with rs174575, together with performance and verbal IQ, finding similar results although effect sizes were generally reduced.

**Conclusions and Significance:**

This study did not replicate the findings of Caspi et al. In contrast to their study, GG children exhibited the greatest difference between feeding methods such that breastfed children performed similarly irrespective of child genotype whereas formula fed GG children performed worse than other children on formula milk. Further studies are required to replicate these findings.

## Introduction

Breast milk uniquely caters for the needs of an infant. It not only serves as a food and a drink but also contains important hormones, enzymes and antibodies. The short and long term benefits of breastfeeding for the child are numerous with one of the most consistently reported findings being for cognitive development [1]. Two recent studies have both concluded benefits for breastfeeding [2], [3]. A meta-analysis of eight studies found breastfed infants scored nearly 5 points higher on standardised intelligence tests. Stronger evidence comes from a recent randomised controlled trial. A benefit of over 7 points for verbal IQ was reported for the intervention group where breastfeeding was promoted compared to controls.

One possible explanation for this beneficial effect may be the differing fatty acid profile between the feeding methods. Breast milk contains arachidonic (AA) and docosahexaenoic acid (DHA) but these fatty acids were absent in most formulas until recently [4], [5]. Of these, DHA is of particular interest due to its concentration in cell membrane phospholipids of the brain [5]–[7]. In addition to maintaining cellular fluidity, DHA has a crucial role in neurogenesis, neurotransmission and protection against oxidative stress [7]. Supporting evidence for the role of DHA in cognitive development has come from animal experiments [8]; from maternal supplementation during pregnancy and lactation [9] although the later benefits may be restricted to particular facets of cognition rather than global IQ [10];from formula supplementation especially in preterm infants [11]; and, from other sources of pre-formed DHA such as fish. Maternal seafood consumption in pregnancy has been associated with more favourable child cognition from ages 6m to 9y [12]–[15].

An alternative strategy is to use genetic variants as proxies for DHA exposure. Such data have advantages being generally resilient to issues of confounding by social and life-style factors and to reverse causation that often influence the results from observational studies [16]. To be useful in terms of providing causal evidence of environmentally modifiable influences on disease outcomes, a polymorphism must be associated with the exposure and unrelated to the outcome other than via its effect on the exposure [17]. The FADS gene family is involved in the desaturation of fatty acids to form the more complex long chain polyunsaturated fatty acids such as DHA. A sequence of delta-5 desaturation associated with the FADS1 gene, delta-6 desaturation associated with the FADS2 gene and elongation by two carbon atoms at the delta or carboxyl terminus metabolises shorter chain fatty acids such as linoleic acid (LA) and α-linolenic acid (ALA) to produce increasingly longer chain and more highly unsaturated fatty acids (see Figure S1). The delta-6 destaturation stages are considered to be the rate-limiting steps in the metabolic process [18]. Minor alleles of variants in the FADS2 gene have been associated with decreased plasma and erythrocyte phospholipid long-chain fatty acid levels in larger studies [19], [20] but not in smaller studies [21], [22], probably reflecting a lack of power in these latter studies. Lower DHA levels in breast milk have also been reported for the maternal GG genotype of rs174575 [5]. These results suggest that minor alleles are associated with lower enzymatic activity and consequently are less efficient at converting precursors to form products. Overall, such genetic variants are likely to be useful in strengthening causal inferences concerning the influence of DHA on health outcomes.

A recent genetic study has shown an interaction between breastfeeding and the rs174575 polymorphism within FADS2 for two different birth cohorts [23]. Children homozygous for the minor allele (G) had comparable IQs irrespective of breastfeeding status, while C carriers (whether homozygous or heterozygous for that allele) demonstrated nearly a 7-point advantage for breastfeeding. This result appeared resilient to potential confounding by gene-environment correlation and differences in intrauterine growth, social class and maternal cognitive ability. Another polymorphism, rs1535 yielded inconsistent findings between the two cohorts. No robust associations were found with maternal genotypes.

This investigation has attempted to replicate these novel findings using data from the Avon Longitudinal Study of Parents and Children (ALSPAC) cohort.

## Methods

### Study population

ALSPAC was established to explore the environmental, social, psychological and genetic factors associated with child health and development. It recruited 14,541 pregnant women in the Bristol area who had an expected delivery date between April 1991 and December 1992. The study area comprises a mixture of rural areas, inner-city deprivation, suburbs and moderate sized towns as well as the city of Bristol. The cohort is broadly similar to the UK in terms of a range of demographic variables. 13,988 children from the study were alive at age one year [24]. Ethical approval for the study was obtained from the ALSPAC Law and Ethics Committee and the Southmead, Frenchay, UBHT and Weston Research Ethics Committees. Written consent was obtained from participants to allow use of anonymized linked genetic data for research by bona fide scientists.

### Outcome

Child IQ was assessed at about age 8y using a short form of the WISC [25]. This measure involves 10 subtests – 5 of which relate to verbal tasks and 5 to performance tasks. Nine of the ten subtests were administered using alternate items. The Coding subtest, which has no stopping rules, was administered in its full form. In all, 1% of children had their summed scaled scores for verbal subtests prorated due to missing one subtest while the equivalent figure was 8% for performance scores.

### FADS2 Genotyping

DNA was extracted from all available stored samples relating to 9656 children and 8678 mothers. Genotyping was undertaken by KBioscience Ltd using their own form of competitive allele specific PCR system (KASPar) for SNP analysis. The failure rates for rs174575 and rs1535 were 6.5% and 5.2% (child); 4.9% and 4.2% (mother). The error rate for genotyping of duplicate samples was <0.2%.

These SNPs were chosen by Caspi et al due to their high linkage disequilibrium with variants throughout the FADS1/FADS2 region and their common minor allele frequency increasing power to detect interactions. These two SNPs accounted for 43% of the genetic variability of the FADS2 gene and 73% if only common variants (minor allele frequency>0.2) are considered (HapMap data release 21, Utah residents with Northern or Western Europe ancestry, see Figure S2).

### Environmental factors

Breastfeeding was the main environmental variable for this investigation. Mothers reported at one month after the birth whether they had ever breastfed during this period. Maternal genotype was also considered. Although it may have direct genetic influences on offspring through inheritance, it may also influence the dietary supply of fatty acids to the offspring both in utero and postnatally via the quality of breast milk [5].

### Statistical analysis

Linear regression was used to analyse full scale, performance and verbal IQ. To assess whether genetic associations were resilient to confounding, adjustment was made in initial analyses for pre-term gestation (<37 w), low birth weight (<2500 g), gender, paternal social class (3 levels: classes I+II, III and IV+V), maternal education (3 levels based upon qualifications obtained: no academic qualifications, academic qualifications at 16 y, academic qualifications at 18 y) and measures of child stimulation both from the home environment and maternal interaction with the child (treated as continuous covariates). Controlling for maternal education (or IQ) and stimulation is considered particularly important [2]. All confounders except gender had strong univariable associations with IQ (p<0.0001) and together explained 14% of the variability in IQ. While genetic main effects are usually unrelated to such factors, gene-environment (GE) interactions may be more sensitive to confounding [26]. Genetic associations were assessed using both an additive per allele model (as supported by the associations with long-chain fatty acids [19], [20]) and a recessive model for the minor allele (as reported by Caspi et al [23]). Gene and breastfeeding main effects were assessed using a hierarchical model. Inheritance effects were assessed for children where maternal transmission of the G allele could be inferred with certainty i.e. for homozygote mothers and children. Paternal transmission was deduced from child genotype and maternal transmission. Main analyses were restricted to those of white ethnic origin. Child ethnicity was either derived from parental ethnicity as reported by the mother or obtained from school census data. Maternal ethnicity used the maternal report or where missing was inferred from white ethnic status for the child. Power calculations suggested this study would have a 95% and 82% chance of detecting the size of the interaction effects observed in the Dunedin and E-risk cohorts at the 5% significance level.

## Results

### FADS2 polymorphisms and Ethnicity (Table 1)

**Table 1:** Allele frequencies and Hardy-Weinberg equilibrium tests for FADS2 polymorphisms by ethnic origin^1^.

		White			Non-white			Missing			Difference
		N	Allele^2^	P^3^	N	Allele^2^	P^3^	N	Allele^2^	P^3^	p 4
Child	rs174575	8282	0.260	0.95	421	0.209	0.025	327	0.249	0.28	0.0011
	rs1535	8393	0.333	0.40	424	0.241	0.0023	336	0.339	0.75	<0.0001
Mother	rs174575	7689	0.258	0.93	163	0.187	0.88	484	0.275	0.41	0.0033
	rs1535	7726	0.333	0.39	162	0.244	0.063	483	0.344	0.78	0.0006

Notes:^1^Maternal ethnic origin was self-reported from a questionnaire administered during pregnancy. Child origin was derived from parental origins reported by the mother and from school census data.

2 Frequencies are for the G allele.

3 HW chi-square test (1df).

4 Difference between allele frequencies for white and non-white groups. Chi-square test (1df).

Within ALSPAC, mothers and children were predominantly classified as white −98% and 95% respectively. Frequencies for the G allele differed by ethnic group for both mothers (p<0.01) and children (p<0.001). Genotypes were in Hardy-Weinberg equilibrium for the white ethnic group but there was some evidence of disequilibria in the non-white group. This may reflect the heterogeneity of that group.

### Breastfeeding and Child IQ

In all, 5934 children of white European origin had genetic data with additional information on breastfeeding and IQ. Of these 83% were breastfed within the first month. These children had means (SDs) of 108 (16), 100 (17) and 105 (16) for verbal, performance and full-scale IQ respectively.

### Gene, Environment and GE effects on Child IQ

As expected, there was no association of genotypes with breastfeeding or confounders (see Table S1).

Breastfeeding showed a strong association with full-scale IQ with breastfed children scoring 8 points higher IQ on average in unadjusted analyses as has been previously reported [2], [3]. There were strong associations between breastfeeding and most confounders (see Table S2). The breastfeeding effect attenuated to a 3-point advantage after adjustment for these confounders (see Table 2).

**Table 2:** Hierarchical linear regression analyses of full-scale IQ with gene x environment effects unadjusted and adjusted for confounders relating to children of white ethnic origin assuming a recessive genetic effect.

		N	Gene^1^				Breastfeeding				Interaction^2^			
			B	95% CI		p	B	95% CI		p	B	95% CI		p
Child	**rs174575**													
	Unadjusted	5045	0.17	−1.57	1.90	0.85	7.80	6.61	8.99	<0.0001	5.78	1.43	10.12	0.0091
	Unadjusted^3^	4411	0.76	−1.09	2.62	0.42	7.75	6.44	9.07	<0.0001	4.70	−0.09	9.50	0.055
	Adjusted^4^	4411	0.76	−0.96	2.49	0.39	3.50	2.21	4.79	<0.0001	4.26	−0.26	8.77	0.065
	**rs1535**													
	Unadjusted	5099	0.10	−1.32	1.51	0.89	7.72	6.54	8.90	<0.0001	4.01	0.36	7.66	0.031
	Unadjusted^3^	4448	0.59	−0.90	2.09	0.44	7.72	6.41	9.02	<0.0001	3.44	−0.59	7.46	0.094
	Adjusted^4^	4448	0.16	−1.23	1.56	0.82	3.48	2.20	4.76	<0.0001	3.71	−0.08	7.50	0.055
Mother	**rs174575**													
	Unadjusted	4026	1.79	−0.23	3.82	0.082	8.26	6.92	9.59	<0.0001	−1.54	−7.43	4.36	0.61
	Unadjusted^3^	3558	1.86	−0.25	3.96	0.084	8.11	6.64	9.58	<0.0001	−0.94	−7.27	5.39	0.77
	Adjusted^4^	3558	1.49	−0.47	3.44	0.14	3.21	1.76	4.66	<0.0001	−2.14	−8.09	3.81	0.48
	**rs1535**													
	Unadjusted	4041	0.76	−0.83	2.34	0.35	8.25	6.92	9.57	<0.0001	0.45	−4.05	4.95	0.84
	Unadjusted^3^	3568	0.87	−0.79	2.53	0.30	8.11	6.65	9.58	<0.0001	1.68	−3.19	6.55	0.50
	Adjusted^4^	3568	0.89	−0.65	2.42	0.26	3.23	1.78	4.67	<0.0001	2.02	−2.56	6.59	0.39

Notes:^1^Recessive effect for minor allele (G).

2 The non-additive effect of breastfeeding and the GG genotype.

3 Restricted sample to match adjusted analyses.

4 Adjusted for maternal education, paternal social class, low birth weight, pre-term gestation, home environment, parenting and gender.

**Figure 1:**
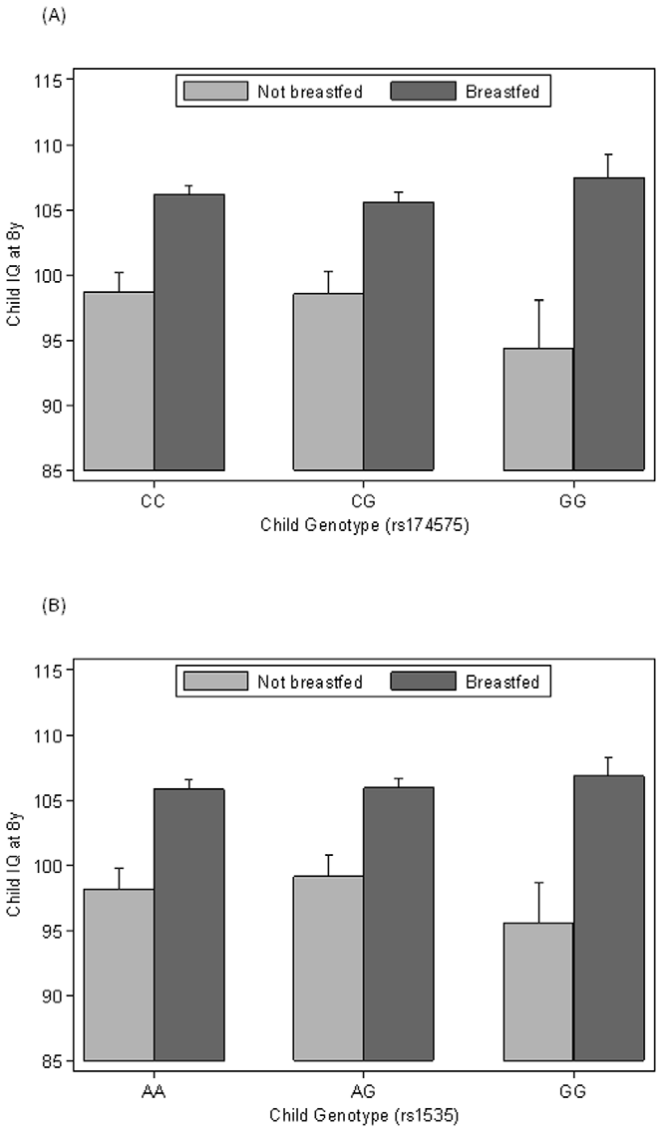
Unadjusted means for Full-scale IQ at 8 years for breastfeeding (yes/no) and child FADS2 genotypes with 95% CIs (N = 5045 (A) and 5099 (B)). Mean IQ scores are calculated for each genotype of the polymorphisms rs174575 (A) and rs1535 (B) with subgroups who were ever breastfed in the first month after birth (black bars) and others not breast fed (grey bars). In addition to the breastfeeding effect (p<0.0001), GG children had particularly low scores when not breastfed (p interaction  = 0.0091 (A) and 0.031 (B)).

IQ was also associated with an interaction between breastfeeding and child genotype. The strongest effects were noted for rs174575 and full-scale IQ although similar effects were observed for rs1535 and for other IQ measures. GG children showed the largest difference between any breastfeeding and exclusive use of formula feeds (see Figure 1). In particular these formula-fed children achieved the lowest scores on average while their breastfed counterparts achieved similar results to other breastfed children carrying the C or A allele (interaction p = 0.0091 for rs174575 and 0.031 for rs1535). This interaction can be interpreted, taking rs174575 as an example, as an association with IQ of −4.3 [95% CI −8.2, −0.4; p = 0.032] points for the GG genotype compared to CC+CG genotypes in formula fed children while breastfed children showed no association (B = 1.5 [95% CI −0.4, 3.4; p = 0.12]). Combining both feeding groups, there was no association with child genotype.

In general, these results were not markedly changed after adjustment (see Table 2). Although sample attrition reduced the significance of results, effect sizes were similar to unadjusted results especially when both analyses were restricted to the same children. But even in this case, some attenuation in the interaction effect size was observed. An estimate of the adjusted interaction effect for rs174575 compensating for attrition was 5.2 [95% CI 0.9, 9.5; p = 0.019] (see Table S3). Repeat analyses using an additive genetic model showed similar results for main effects but there was no evidence of a GE interaction (see Table S4).

Maternal genotype was not associated with child IQ in univariable analyses. No interaction with breastfeeding was observed.

Further analyses explored the effect of inheritance amongst formula fed children where a genetic association was observed. There was no evidence of a differential parental inheritance effect on IQ (p = 0.20 for rs174575 and 0.21 for rs1535).

### Haplotypes

The two SNPs showed evidence of linkage disequilibrium (Delta statistic  = 0.81 for both mother and offspring). Analysis of the GG child haplotype showed no evidence of an interaction with breastfeeding if a linear dose effect was assumed for 0, 1 or 2 copies (p = 0.14). However if an effect was assumed solely for 2 copies, strong evidence for an interaction existed (p = 0.0036). Other haplotypes showed no association with child IQ.

## Discussion

This investigation has studied the role of two SNPs within the FADS2 gene and breastfeeding on child IQ in an attempt to replicate the findings from two cohorts reported by Caspi et al [23]. All three cohorts reported a large breastfeeding effect for CC and CG genotypes. In addition they all suggested that the GG genotype modified the effect of breastfeeding but in this case the direction of the effects differed. Whereas Caspi et al reported no breastfeeding effect for GG children on rs174575 in two cohorts (N = 858 and 1848), this study (N = 5045) showed a larger effect than observed for other genotypes. Perhaps not surprisingly, a meta-analysis of the interaction effect for rs174575 showed heterogeneity between the cohorts (I^2^ = 88%, p = 0.0002) with a null pooled estimate of −0.9 (95% CI −3.9, 2.0; p = 0.54). (The negative effect reflects an attenuation of the breastfeeding effect for GG children.) A number of possible reasons might explain this variation in results.

One explanation may be that these differing results reflect inter-study differences. While all studies consisted mainly of European-origin populations, allele frequencies suggested the two UK studies (ALSPAC and E-risk) were genetically more similar than the New Zealand study (26, 26, 30% and 33, 34, 39% for the three studies and rs174575 and rs1535 respectively). However, this pattern of differences and similarities was not reflected in the results. There was some evidence that the correlation between breastfeeding and other factors differed between the studies. There were strong associations of breastfeeding with birth weight and gestation in this study with inconsistent or null results reported for the other two cohorts. There could be other important differences between the studies such as the extent by which breastfeeding may affect child dietary preferences in later life. Such changes may alter the supply of pre-formed DHA or alternatively alter the omega-3 to omega-6 ratio. This ratio is important since both these types of polyunsaturated fatty acids compete for the same enzyme encoded by FADS2. Consequently an excess of omega-6 fatty acids may limit the synthesis of omega-3 fatty acids such as DHA. Whether any of these observed or hypothesised differences could explain the discordant results remains unclear. Analyses in this study suggested that this explanation might be unlikely since adjustment had a much smaller attenuation on the interaction (about 10%) compared to the breastfeeding main effect (over 50%). The other two cohorts did not perform multivariable adjustment for such factors relying on null univariable associations to infer a null cumulative effect on the interaction.

Another explanation could involve the definition of breastfeeding. In the Dunedin and E-risk studies, data were collected retrospectively at ages 2 or 3 years and may be subject to recall bias especially when this feeding method was used for a short period. By contrast, this study collected information prospectively. It should be noted that the breastfed children reported here included 20% who had changed to exclusive use of formula during the first month. This in part explains the varying prevalence of breastfeeding across the studies. Reclassifying the short-term breastfed children as formula fed in this study generally produced the same pattern of results albeit with the interaction effect attenuated. While this study's results were relatively robust to misclassification, other studies may be more sensitive.

A third explanation could be that some or all results reflect chance events as the result of intra-study variability. False-positive claims from genetic association studies have until recently been the norm reflecting the very large number of associations that can be studied, the small effect sizes that can be anticipated, the low power of studies against this background and publication bias [27]. While results in opposite directions are consistent with this hypothesis, it is an unusual scenario that none of the three results were null. Replication is clearly essential [28]. In this regard, it is interesting to note that an initial report of an interaction between a variant of the serotonin transporter gene and stressful life events on depression [29] was not consistently replicated in later studies [30].

While further studies may resolve the contrasting results so far obtained, it may be useful to explore the plausibility of these results and the possible underlying mechanisms. Breast milk is a complex food providing numerous benefits [1]. It should be of benefit to all children including those with GG genotype. This makes the results from the Caspi study of no breastfeeding effect for GG children difficult to interpret. In contrast, this study found a large breastfeeding effect for all genotypes. A possible mechanism for our results may lie in the concept of nutritional adequacy whereby no additional benefit is achieved once an individual's nutrient requirement is met [6]. Breast milk contains pre-formed DHA and consequently does not require the metabolism of other fatty acids via elongation and desaturation [5]. This supply may be sufficient to meet infant needs irrespective of FADS genotype. This would be reflected in similar IQs as observed. On the other hand, although formula contains α-linolenic acid (ALA) and for some brands in similar quantities to breast milk [4], GG children would be less able to convert this to DHA [20]. Since, as in the Caspi study, no families used formula enriched with DHA or AA, the combination of formula and GG genotype would appear consistent with nutritional deficiency as a result of inadequate supply and synthesis. This explanation is compatible with our result of the lowest IQs on average for these children. Other genotypes for formula fed children would be increasingly able to synthesise DHA from ALA. Our results suggest that heterozygotes are able to achieve nutritional adequacy. Since under this model, only formula fed GG children are nutritionally deficient in DHA, the breastfeeding effect for the other genotypes should be attributed to other, non-DHA related, benefits of breast milk or the effect of confounding factors. This model appears appropriate and helps to explain why interaction effects were only observed using a recessive genetic effect rather than the additive effect associated with fatty acids [19], [20].

A recent article has suggested that GE interactions may imply genetic plasticity rather than genetic vulnerability [31]. This theory suggests that one genotype may not only infer a vulnerability to environmental deprivation but also be more responsive to a favourable environment. While GG individuals appear to exhibit some of these features, such that these children had the lowest IQs on average amongst non-breastfed children (−4.27 [95% CI −7.89, −0.64]; p = 0.021) and the highest IQs amongst breastfed (1.51 [−0.41, 3.43]; p = 0.12), the lack of statistical evidence for the latter result may favour the diathesis–stress framework over plasticity.

This study also found similar effects for rs1535 although the results were less robust. Caspi et al. reported inconsistent results for this SNP between their two studies.

Similar to the Caspi study, no associations were observed with the maternal genotype. Although the quality of breast milk changes with maternal genotype [5], this was not reflected in changes in IQ at 8y. In addition, the maternal genotype is likely to affect the supply of fatty acids to the fetus [5], [32]. Such effects might be expected to be most evident in the formula fed children but none were observed.

It is interesting to note that studies of maternal fish consumption in pregnancy have tended to show the strongest associations for verbal rather than full-scale IQ [13], [33]. But in this study, in regard to breastfeeding, the reverse was generally true although sometimes the differences were marginal. It is possible that the different timing of exposure affects different developmental stages in the brain.

A limitation of this study was the risk of bias due to the lack of controlling for other potentially important factors such as maternal IQ [34], maternal fish consumption during lactation and the ratio of omega-6 to omega-3 fatty acids. These data were not available within ALSPAC. However data on maternal educational qualifications were available. This measure is likely to be highly correlated with maternal IQ and more strongly related than other measures of education such as years of schooling. In addition, adjustment for educational qualifications did not change the conclusions despite being an important predictor of child IQ (p<0.001). DHA in breast milk is affected by fish consumption and fish oil supplementation [35], [36]. It is possible that the GE interaction may reflect mothers of breastfed GG children consuming more fish than other mothers in this study (or less fish in the Dunedin and E-risk studies). But use of maternal fish consumption during pregnancy as a proxy for consumption during lactation only changed the results by about 1%. The ratio of omega-6 to omega-3 fatty acids might affect the metabolism of DHA from ALA. This will be of particular importance to formula fed infants. While other evidence suggests that this ratio can be similar to breast milk, the most popular brand of formula used by mothers in this study had a higher omega-6 content [4]. Analysis of five brands of formula showed no differences in their effects on child IQ (p = 0.46). This would seem to suggest that either the impact of changes in this ratio was minor or its effect on IQ did not persist to 8 years. Overall, while residual confounding due to these or other factors might be expected to further attenuate the interaction effect, the above comments and the extent of the attenuation of the breastfeeding effect due to the 7 factors used in this study may suggest that the main sources of confounding have been accounted for.

An additional limitation of this study was the impact of sample attrition on the adjusted analyses. These analyses produced less robust evidence of gene-breastfeeding interactions (eg p = 0.065 for rs174575) with some ambiguity whether this should be interpreted as the interaction being totally due to confounding or that an attenuated interaction effect was now below the level of detection in the smaller sample. Supplementary analyses, which attempted to compensate for attrition, favoured this latter explanation. Nevertheless, even in this large study, the CIs for the interaction effect were wide. As a consequence, the observed effect was consistent, at one extreme, with a small additional loss of one IQ point for GG children fed formula compared to their breastfed counterparts but, at the other extreme, a loss of 10 IQ points reflecting a difference exceeding the main breastfeeding effect.

In summary, this investigation found that GG children had the lowest average IQs amongst formula fed children, but when breastfed, their scores were similar to CC and CG children. These results differ from the findings of Caspi et al [23] and require replication. Until a greater consensus across studies is achieved and with the potential risk of bias to GE interactions from the observational nature of this study [26], we caution against over-interpretation of these results despite a plausible biological mechanism being described.

## Supporting Information

Table S1 Associations of breastfeeding and confounders with FADS2 genotypes
(0.08 MB DOC)

Table S2 Association of confounders with breastfeeding
(0.03 MB DOC)

Table S3 Investigation of the effect of sample attrition on the breastfeeding-rs174575 interaction effect in adjusted analyses of Full Scale IQ
(0.04 MB DOC)

Table S4 Hierarchical linear regression analyses of full-scale IQ with gene x environment effects unadjusted and adjusted for confounders relating to children of white ethnic origin assuming an additive genetic effect
(0.06 MB DOC)

Figure S1 The metabolic pathway of omega-6 and omega-3 fatty acids. Fatty acids are shown with their common/scientific name, their abbreviated name in parentheses and their chemical structure (length of carbon chain: number of unsaturated bonds). LA and ALA are essential fatty acids and cannot be synthesised by humans. Their primary role is as pre-cursors to the more biologically active AA and EPA involved in the production of eicosanoids and DHA associated with docosanoids. Metabolic stages involve delta-5 (Δ5) and delta-6 (Δ6) desaturation associated with FADS1 and FADS2 genes respectively, elongation (E) and beta-oxidation (β).
(0.03 MB PDF)

Figure S2 Linkage Disequilibrium map of variants in the FADS2 gene. The figure shows linkage disequilibria (D') for 26 variants of the FADS2 gene (part A) and for a subset of 13 common variants (minor allele frequency >0.2) (part B). The two polymorphisms, rs1535 and rs174575, are shown in boxes. Data source: HapMap release 21, CEPH database.
(0.13 MB PDF)

## References

[1] Hoddinott P, Tappin D, Wright C (2008). Breast feeding.. BMJ.

[2] Horta BL, Bahl R, Martines JC, Victora CG (2007). Evidence on the long-term effects of breastfeeding: systematic reviews and meta-analyses..

[3] Kramer MS, Aboud F, Mironova E, Vanilovich I, Platt RW (2008). Breastfeeding and child cognitive development: new evidence from a large randomized trial.. Arch Gen Psychiatry.

[4] Ministry of Agriculture Fisheries and Food (1998). Fatty Acids: Seventh Supplement to the Fifth Edition of McCance and Widdowson's The Composition of Foods..

[5] Xie L, Innis SM (2008). Genetic variants of the FADS1 FADS2 gene cluster are associated with altered (n-6) and (n-3) essential fatty acids in plasma and erythrocyte phospholipids in women during pregnancy and in breast milk during lactation.. J Nutr.

[6] Food and Nutrition Board (2005). Dietary reference intakes for energy, carbohydrate, fiber, fat, fatty acids, cholesterol, protein, and amino acids..

[7] Innis SM (2007). Dietary (n-3) fatty acids and brain development.. J Nutr.

[8] Fedorova I, Salem N (2006). Omega-3 fatty acids and rodent behavior.. Prostaglandins Leukot Med.

[9] Helland IB, Smith L, Saarem K, Saugstad OD, Drevon CA (2003). Maternal supplementation with very-long-chain n-3 fatty acids during pregnancy and lactation augments children's IQ at 4 years of age.. Pediatrics.

[10] Helland IB, Smith L, Blomen B, Saarem K, Saugstad OD (2008). Effect of supplementing pregnant and lactating mothers with n-3 very-long-chain fatty acids on children's IQ and body mass index at 7 years of age.. Pediatrics.

[11] Fleith M, Clandinin MT (2005). Dietary PUFA for preterm and term infants: Review of clinical studies.. Crit Rev Food Sci Nutr.

[12] Gale CR, Robinson SM, Godfrey KM, Law CM, Schlotz W (2008). Oily fish intake during pregnancy - association with lower hyperactivity but not with higher full-scale IQ in offspring.. J Child Psychol Psychiatry.

[13] Hibbeln JR, Davis JM, Steer C, Emmett P, Rogers I (2007). Maternal seafood consumption in pregnancy and neurodevelopmental outcomes in childhood (ALSPAC study): an observational cohort study.. Lancet.

[14] Oken E, Radesky JS, Wright RO, Bellinger DC, Amarasiriwardena CJ (2008). Maternal fish intake during pregnancy, blood mercury levels, and child cognition at age 3 years in a US cohort.. Am J Epidemiol.

[15] Oken E, Wright RO, Kleinman KP, Bellinger D, Amarasiriwardena CJ (2005). Maternal fish consumption, hair mercury, and infant cognition in a U.S. Cohort.. Environ Health Perspect.

[16] Davey Smith G, Lawlor DA, Harbord R, Timpson N, Day I (2007). Clustered environments and randomized genes: A fundamental distinction between conventional and genetic epidemiology.. PLoS Med.

[17] Davey Smith G, Ebrahim S (2003). ‘Mendelian randomization’: can genetic epidemiology contribute to understanding environmental determinants of disease?. Int J Epidemiol.

[18] Sprecher H (2000). Metabolism of highly unsaturated n-3 and n-6 fatty acids.. Biochimica Et Biophysica Acta-Molecular and Cell Biology of Lipids.

[19] Schaeffer L, Gohlke H, Muller M, Heid IM, Palmer LJ (2006). Common genetic variants of the FADS1 FADS2 gene cluster and their reconstructed haplotypes are associated with the fatty acid composition in phospholipids.. Hum Mol Genet.

[20] Tanaka T, Shen J, Abecasis GR, Kisialiou A, Ordovas JM (2009). Genome-wide association study of plasma polyunsaturated fatty acids in the InCHIANTI Study.. PLoS Genet.

[21] Rzehak P, Heinrich J, Klopp N, Schaeffer L, Hoff S (2009). Evidence for an association between genetic variants of the fatty acid desaturase 1 fatty acid desaturase 2 (FADS1 FADS2) gene cluster and the fatty acid composition of erythrocyte membranes.. Br J Nutr.

[22] Gieger C, Geistlinger L, Altmaier E, de Angelis MH, Kronenberg F (2008). Genetics Meets Metabolomics: A Genome-Wide Association Study of Metabolite Profiles in Human Serum.. PLoS Genet.

[23] Caspi A, Williams B, Kim-Cohen J, Craig IW, Milne BJ (2007). Moderation of breastfeeding effects on the IQ by genetic variation in fatty acid metabolism.. Proc Natl Acad Sci U S A.

[24] Golding J, Pembrey M, Jones R, the ALSPAC Study Team (2001). ALSPAC – the Avon Longitudinal Study of Parents and Children: I. Study methodology.. Paediatr Perinat Epidemiol.

[25] Wechsler D, Golombok S, Rust J (1992). Wechsler Intelligence Scale for Children - Third Edition UK..

[26] Davey Smith G, Ebrahim S, Weinstein M, Vaupel JW, Wachter KW (2008). Mendelian Randomization: Genetic Variants as Instruments for Strengthening Causal Inference in Observational Studies.. Biosocial Surveys: Committee on Advances in Collecting and Utilizing Biological Indicators and Genetic Information in Social Science Surveys.

[27] Colhoun HM, McKeigue PM, Davey Smith G (2003). Problems of reporting genetic associations with complex outcomes.. Lancet.

[28] Chanock SJ, Manolio T, Boehnke M, Boerwinkle E, Hunter DJ (2007). Replicating genotype-phenotype associations.. Nature.

[29] Caspi A, Sugden K, Moffitt TE, Taylor A, Craig IW (2003). Influence of life stress on depression: Moderation by a polymorphism in the 5-HTT gene.. Science.

[30] Risch N, Herrell R, Lehner T, Liang KY, Eaves L (2009). Interaction between the serotonin transporter gene (5-HTTLPR), stressful life events, and risk of depression: a meta-analysis.. JAMA.

[31] Belsky J, Jonassaint C, Pluess M, Stanton M, Brummett B (2009). Vulnerability genes or plasticity genes?. Mol Psychiatry.

[32] Donahue SMA, Rifas-Shiman SL, Olsen SF, Gold DR, Gillman MW (2009). Associations of maternal prenatal dietary intake of n-3 and n-6 fatty acids with maternal and umbilical cord blood levels.. Prostaglandins Leukot Med.

[33] Gale CR, Robinson SM, Godfrey KM, Law CM, Schlotz W (2008). Oily fish intake during pregnancy - association with lower hyperactivity but not with higher full-scale IQ in offspring.. J Child Psychol Psychiatry.

[34] Der G, Batty GD, Deary IJ (2006). Effect of breast feeding on intelligence in children: prospective study, sibling pairs analysis, and meta-analysis.. BMJ.

[35] Lauritzen L, Jorgensen MH, Hansen HS, Michaelsen KF (2002). Fluctuations in human milk long-chain PUFA levels in relation to dietary fish intake.. Lipids.

[36] Dunstan JA, Mitoulas LR, Dixon G, Doherty DA, Hartmann PE (2007). The effects of fish oil supplementation in pregnancy on breast milk fatty acid composition over the course of lactation: a randomized controlled trial.. Pediatr Res.

