# Supplementary material for: FADS2 Polymorphisms Modify the Effect of Breastfeeding on Child IQ
Source: PLoS One. 2010 Jul 13;5(7):e11570. doi: 10.1371/journal.pone.0011570 (PMC2903485; doi:10.1371/journal.pone.0011570)
Supplement: Table S1 — Associations of breastfeeding and confounders with FADS2 genotypes (0.08 MB DOC) [file pone.0011570.s001.doc]

Table S1: Associations of breastfeeding and confounders with FADS2 genotypes

| Outcomes | CC or AA | | CG or AG | | GG | | p | N |
| --- | --- | --- | --- | --- | --- | --- | --- | --- |
|  | Mean | SD | Mean | SD | Mean | SD |  |  |
| Child rs174575 |  |  |  |  |  |  |  |  |
| Breastfeeding | 0.84 | 0.36 | 0.83 | 0.37 | 0.81 | 0.40 | 0.23 | 5045 |
| Maternal education | 2.27 | 0.76 | 2.25 | 0.76 | 2.28 | 0.77 | 0.69 | 4411 |
| Social class | 2.40 | 0.66 | 2.41 | 0.67 | 2.38 | 0.71 | 0.84 | 4411 |
| Gender | 1.50 | 0.50 | 1.50 | 0.50 | 1.50 | 0.50 | 0.96 | 4411 |
| Pre-term birth | 0.05 | 0.22 | 0.05 | 0.21 | 0.04 | 0.21 | 0.79 | 4411 |
| Low birthweight | 0.04 | 0.19 | 0.04 | 0.20 | 0.05 | 0.21 | 0.63 | 4411 |
| HOME score | 8.25 | 2.16 | 8.16 | 2.19 | 8.29 | 2.22 | 0.37 | 4411 |
| Parenting | 10.52 | 1.48 | 10.45 | 1.52 | 10.53 | 1.52 | 0.26 | 4411 |
| Child rs1535 |  |  |  |  |  |  |  |  |
| Breastfeeding | 0.84 | 0.37 | 0.84 | 0.37 | 0.82 | 0.38 | 0.51 | 5099 |
| Maternal education | 2.25 | 0.75 | 2.25 | 0.76 | 2.32 | 0.76 | 0.10 | 4448 |
| Social class | 2.39 | 0.67 | 2.42 | 0.66 | 2.40 | 0.69 | 0.27 | 4448 |
| Gender | 1.51 | 0.50 | 1.49 | 0.50 | 1.48 | 0.50 | 0.33 | 4448 |
| Pre-term birth | 0.05 | 0.22 | 0.05 | 0.22 | 0.05 | 0.21 | 0.92 | 4448 |
| Low birthweight | 0.04 | 0.19 | 0.04 | 0.20 | 0.04 | 0.20 | 0.71 | 4448 |
| HOME score | 8.20 | 2.17 | 8.22 | 2.18 | 8.28 | 2.17 | 0.76 | 4448 |
| Parenting | 10.48 | 1.51 | 10.53 | 1.48 | 10.54 | 1.45 | 0.60 | 4448 |
| Mother rs174575 |  |  |  |  |  |  |  |  |
| Breastfeeding | 0.84 | 0.37 | 0.82 | 0.38 | 0.87 | 0.34 | 0.11 | 4110 |
| Maternal education | 2.25 | 0.76 | 2.26 | 0.76 | 2.28 | 0.78 | 0.76 | 3615 |
| Social class | 2.40 | 0.66 | 2.35 | 0.69 | 2.44 | 0.67 | 0.063 | 3615 |
| Gender | 1.50 | 0.50 | 1.50 | 0.50 | 1.51 | 0.50 | 0.86 | 3615 |
| Pre-term birth | 0.05 | 0.21 | 0.04 | 0.21 | 0.06 | 0.24 | 0.59 | 3615 |
| Low birthweight | 0.04 | 0.20 | 0.03 | 0.18 | 0.04 | 0.21 | 0.30 | 3615 |
| HOME score | 8.20 | 2.20 | 8.18 | 2.11 | 7.92 | 2.26 | 0.13 | 3615 |
| Parenting | 10.52 | 1.45 | 10.56 | 1.46 | 10.38 | 1.52 | 0.089 | 3615 |
| Mother rs1535 |  |  |  |  |  |  |  |  |
| Breastfeeding | 0.83 | 0.37 | 0.82 | 0.38 | 0.86 | 0.35 | 0.14 | 4126 |
| Maternal education | 2.27 | 0.75 | 2.25 | 0.77 | 2.25 | 0.77 | 0.77 | 3626 |
| Social class | 2.40 | 0.67 | 2.37 | 0.68 | 2.41 | 0.67 | 0.29 | 3626 |
| Gender | 1.51 | 0.50 | 1.49 | 0.50 | 1.49 | 0.50 | 0.65 | 3626 |
| Pre-term birth | 0.05 | 0.21 | 0.04 | 0.21 | 0.05 | 0.23 | 0.70 | 3626 |
| Low birthweight | 0.04 | 0.20 | 0.03 | 0.17 | 0.04 | 0.20 | 0.19 | 3626 |
| HOME score | 8.17 | 2.20 | 8.20 | 2.09 | 7.98 | 2.34 | 0.24 | 3626 |
| Parenting | 10.50 | 1.47 | 10.58 | 1.42 | 10.41 | 1.53 | 0.084 | 3626 |

Ordinal regression was used to estimate significance of genotype effects for outcomes (breastfeeding and confounders). The sample was restricted to those children of white ethnic origin with Full Scale IQ for breastfeeding and those with complete data for confounders.
