# Supplementary material for: FADS2 Polymorphisms Modify the Effect of Breastfeeding on Child IQ
Source: PLoS One. 2010 Jul 13;5(7):e11570. doi: 10.1371/journal.pone.0011570 (PMC2903485; doi:10.1371/journal.pone.0011570)
Supplement: Table S2 — Association of confounders with breastfeeding (0.03 MB DOC) [file pone.0011570.s002.doc]

Table S2: Association of confounders with breastfeeding

| Outcomes | Non-breastfed | | Breastfed | | p |
| --- | --- | --- | --- | --- | --- |
|  | Mean | SD | Mean | SD |  |
| Maternal education | 1.83 | 0.72 | 2.34 | 0.74 | <0.0001 |
| Social class | 2.13 | 0.69 | 2.45 | 0.65 | <0.0001 |
| Gender | 1.49 | 0.50 | 1.50 | 0.50 | 0.63 |
| Pre-term birth | 0.09 | 0.29 | 0.04 | 0.20 | <0.0001 |
| Low birthweight | 0.07 | 0.26 | 0.03 | 0.18 | <0.0001 |
| HOME score | 7.86 | 2.22 | 8.28 | 2.16 | <0.0001 |
| Parenting | 10.15 | 1.68 | 10.56 | 1.45 | <0.0001 |

Ordinal regression was used to estimate significance of breastfeeding effects for outcomes (confounders). The sample was restricted to those children of white ethnic origin with Full Scale IQ and rs174575 data (N=4411).

Maternal education: 1= none or vocational qualifications, 2=ordinary level qualification usually taken at age 16 years, 3=advanced level qualification usually obtained at age 18 years or degree.

Social class relating to father’s occupation: 1= classes IV and V, 2=class III, 3= classes I and II.

Gender: 1=male, 2= female.

Pre-term birth: 0= full term, 1= pre-term (<37w)

Low birth weight: 0= normal, 1= low (<2500g)
