# Supplementary material for: FADS2 Polymorphisms Modify the Effect of Breastfeeding on Child IQ
Source: PLoS One. 2010 Jul 13;5(7):e11570. doi: 10.1371/journal.pone.0011570 (PMC2903485; doi:10.1371/journal.pone.0011570)
Supplement: Table S3 — Investigation of the effect of sample attrition on the breastfeeding-rs174575 interaction effect in adjusted analyses of Full Scale IQ (0.04 MB DOC) [file pone.0011570.s003.doc]

Table S3: Investigation of the effect of sample attrition on the breastfeeding-rs174575 interaction effect in adjusted analyses of Full Scale IQ

| Confounder | Maximum data | | | | Complete data (N=4411) | | |
| --- | --- | --- | --- | --- | --- | --- | --- |
|  | N | B1 | B2 | B1/B2 | B1 | B2 | B1/B2 |
| Maternal education | 4937 | 5.225 | 5.441 | 0.960 | 4.542 | 4.703 | 0.966 |
| Social class | 4616 | 4.658 | 4.775 | 0.975 | 4.497 | 4.703 | 0.956 |
| Gender | 5045 | 5.789 | 5.776 | 1.002 | 4.709 | 4.703 | 1.001 |
| Pre-term birth | 5045 | 5.812 | 5.776 | 1.006 | 4.736 | 4.703 | 1.007 |
| Low birth weight | 4987 | 5.784 | 5.918 | 0.977 | 4.640 | 4.703 | 0.987 |
| HOME score | 4851 | 5.328 | 5.474 | 0.973 | 4.492 | 4.703 | 0.955 |
| Parenting | 4841 | 5.499 | 5.494 | 1.001 | 4.661 | 4.703 | 0.991 |
| All confounders |  |  |  |  | 4.256 | 4.703 | 0.905 |
| Cumulative effect |  |  |  | 0.900 |  |  | 0.870 |

The table shows the effect of each confounder individually on the interaction effect with B1 being the regression coefficient adjusted for the confounder and B2 being the equivalent unadjusted result for the same children. These results are shown for the maximum data available and for the restricted sample with complete data on all the confounders. The ratio B1/B2 is a measure of the attenuation effect due to adjustment. The cumulative attention effect, assuming all confounders are independent, is the product of the individual attenuation effects. In practice, this estimate of the cumulative effect exaggerated the true effect ie –13.0% compared to the observed cumulative effect –9.5%. Furthermore, as the sample size increases, there was a tendency for the attenuation percentage to decrease reflecting the larger interaction effect observed. As a consequence, we might expect the cumulative effect to move closer to 1 as the average sample size of 4903 for maximum data approaches the full sample of 5045. For these two reasons, it is likely that using an attenuation of 10% would overestimate the true attenuation for the full sample.

In addition to attenuation of effect sizes, adjustment also reduced the residual variance. Hence, the SE of the interaction effect reduced from 2.445 without confounders to 2.302 with confounders (N=4411) – a reduction of 6%.

A third effect of adjustment was to reduce the sample size with not only an impact on power but also the possibility that the 634 children excluded were in some way different from the 4411 with complete data on confounders. Although the unadjusted interaction effects were somewhat different numerically (B=9.271 for the excluded children, B=4.703 for the included children), a comparison of the effects for the two groups indicated they were equivalent (p=0.43).

Taken together, these results suggest the original unadjusted result of 5.776 (SE=2.215) for the full sample might be corrected to 5.198 (2.085) with p=0.013 upon adjustment. A more conservative estimate of the significance assuming confounders do not reduce the residual variance would be p=0.019.
