# Supplementary material for: FADS2 Polymorphisms Modify the Effect of Breastfeeding on Child IQ
Source: PLoS One. 2010 Jul 13;5(7):e11570. doi: 10.1371/journal.pone.0011570 (PMC2903485; doi:10.1371/journal.pone.0011570)
Supplement: Table S4 — Hierarchical linear regression analyses of full-scale IQ with gene x environment effects unadjusted and adjusted for confounders relating to children of white ethnic origin assuming an additive genetic effect (0.06 MB DOC) [file pone.0011570.s004.doc]

Table S4: Hierarchical linear regression analyses of full-scale IQ with gene x environment effects unadjusted and adjusted for confounders relating to children of white ethnic origin assuming an additive genetic effect.

|  |  | N | Gene^1^ | | | | Breastfeeding | | | | Interaction^2^ | | | |
| --- | --- | --- | --- | --- | --- | --- | --- | --- | --- | --- | --- | --- | --- | --- |
|  |  |  | B | 95% CI | | p | B | 95% CI | | p | B | 95% CI | | p |
| Child | **rs174575** |  |  |  |  |  |  |  |  |  |  |  |  |  |
|  | Unadjusted | 5045 | -0.27 | -0.99 | 0.44 | 0.45 | 7.78 | 6.59 | 8.98 | <0.0001 | 1.40 | -0.46 | 3.25 | 0.14 |
|  | Unadjusted^3^ | 4411 | -0.09 | -0.85 | 0.67 | 0.82 | 7.74 | 6.42 | 9.05 | <0.0001 | 0.91 | -1.14 | 2.96 | 0.38 |
|  | Adjusted^4^ | 4411 | -0.01 | -0.72 | 0.69 | 0.97 | 3.48 | 2.19 | 4.77 | <0.0001 | 0.76 | -1.16 | 2.69 | 0.44 |
|  | **rs1535** |  |  |  |  |  |  |  |  |  |  |  |  |  |
|  | Unadjusted | 5099 | 0.18 | -0.49 | 0.85 | 0.60 | 7.72 | 6.54 | 8.90 | <0.0001 | 1.00 | -0.75 | 2.75 | 0.26 |
|  | Unadjusted^3^ | 4448 | 0.24 | -0.47 | 0.94 | 0.51 | 7.71 | 6.40 | 9.02 | <0.0001 | 0.82 | -1.10 | 2.75 | 0.40 |
|  | Adjusted^4^ | 4448 | 0.04 | -0.62 | 0.69 | 0.92 | 3.47 | 2.19 | 4.76 | <0.0001 | 1.00 | -0.81 | 2.81 | 0.28 |
| Mother | **rs174575** |  |  |  |  |  |  |  |  |  |  |  |  |  |
|  | Unadjusted | 4026 | 0.66 | -0.16 | 1.48 | 0.11 | 8.28 | 6.95 | 9.62 | <0.0001 | 0.62 | -1.61 | 2.85 | 0.59 |
|  | Unadjusted^3^ | 3558 | 0.66 | -0.20 | 1.52 | 0.13 | 8.13 | 6.66 | 9.60 | <0.0001 | 1.13 | -1.31 | 3.56 | 0.37 |
|  | Adjusted^4^ | 3558 | 0.61 | -0.19 | 1.41 | 0.13 | 3.23 | 1.78 | 4.68 | <0.0001 | 0.58 | -1.71 | 2.87 | 0.62 |
|  | **rs1535** |  |  |  |  |  |  |  |  |  |  |  |  |  |
|  | Unadjusted | 4041 | 0.47 | -0.28 | 1.22 | 0.22 | 8.25 | 6.92 | 9.57 | <0.0001 | 0.54 | -1.50 | 2.58 | 0.61 |
|  | Unadjusted^3^ | 3568 | 0.40 | -0.39 | 1.19 | 0.32 | 8.12 | 6.66 | 9.58 | <0.0001 | 1.18 | -1.05 | 3.42 | 0.30 |
|  | Adjusted^4^ | 3568 | 0.47 | -0.26 | 1.21 | 0.21 | 3.23 | 1.79 | 4.67 | <0.0001 | 1.12 | -0.98 | 3.22 | 0.29 |

Notes: ^1^ Additive effect for minor allele (G)

^2^ The non-additive effect of breastfeeding and the GG genotype.

^3^ Restricted sample to match adjusted analyses.

^4^ Adjusted for maternal education, paternal social class, low birth weight, pre-term gestation, home environment, parenting and gender.
