# Supplementary figures and images for: FADS2 Polymorphisms Modify the Effect of Breastfeeding on Child IQ
Source: PLoS One. 2010 Jul 13;5(7):e11570. doi: 10.1371/journal.pone.0011570 (PMC2903485; doi:10.1371/journal.pone.0011570)

## OMEGA-6 FATTY ACIDS

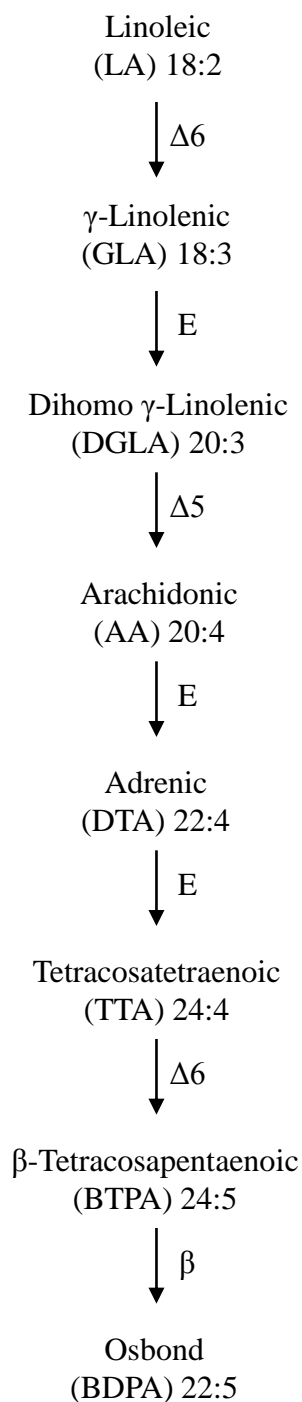

## OMEGA-3 FATTY ACIDS

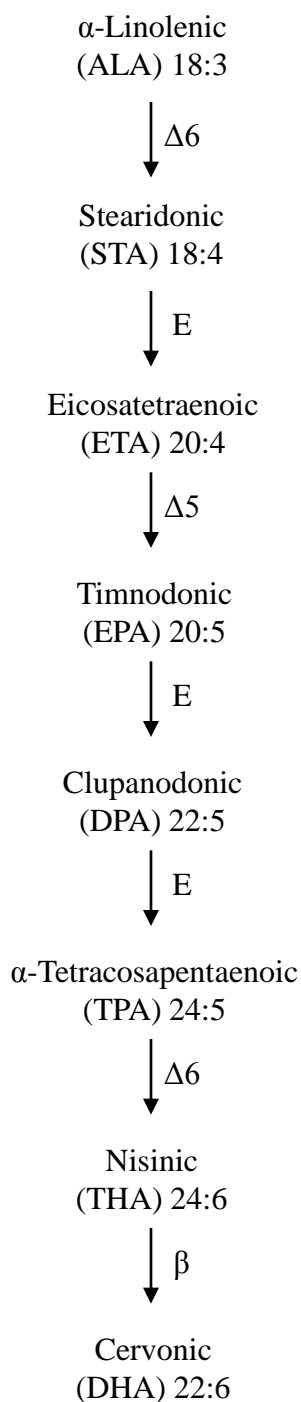

Supplement: Figure S1 — The metabolic pathway of omega-6 and omega-3 fatty acids. Fatty acids are shown with their common/scientific name, their abbreviated name in parentheses and their chemical structure (length of carbon chain: number of unsaturated bonds). LA and ALA are essential fatty acids and cannot be synthesised by humans. Their primary role is as pre-cursors to the more biologically active AA and EPA involved in the production of eicosanoids and DHA associated with docosanoids. Metabolic stages involve delta-5 (Δ5) and delta-6 (Δ6) desaturation associated with FADS1 and FADS2 genes respectively, elongation (E) and beta-oxidation (β). (0.03 MB PDF) [file pone.0011570.s005.pdf]

(A)

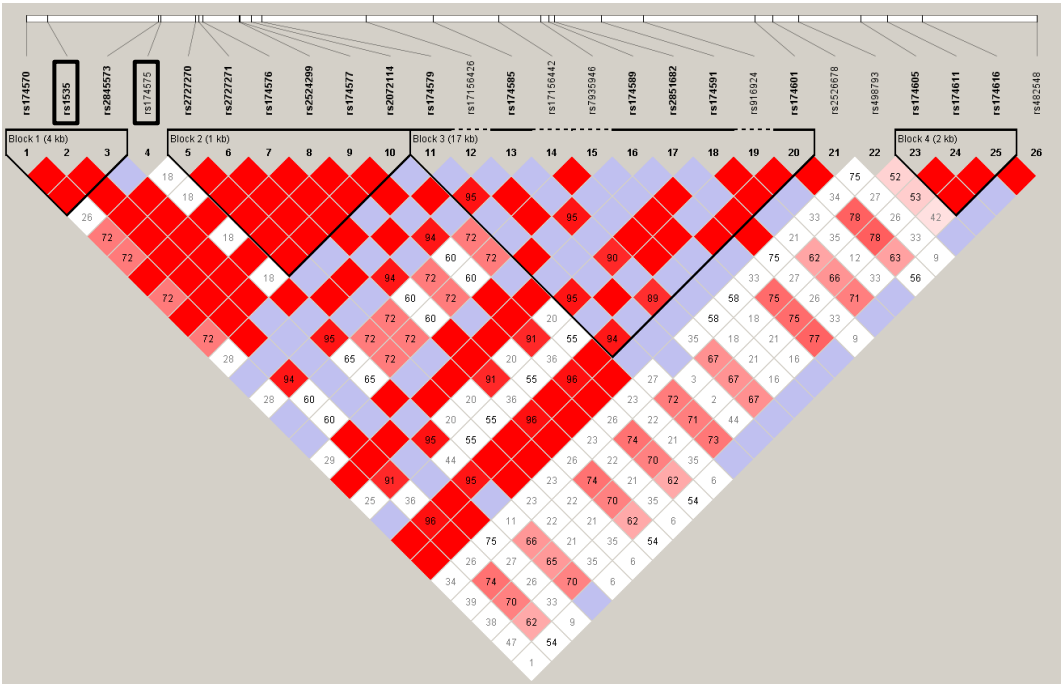

(B)

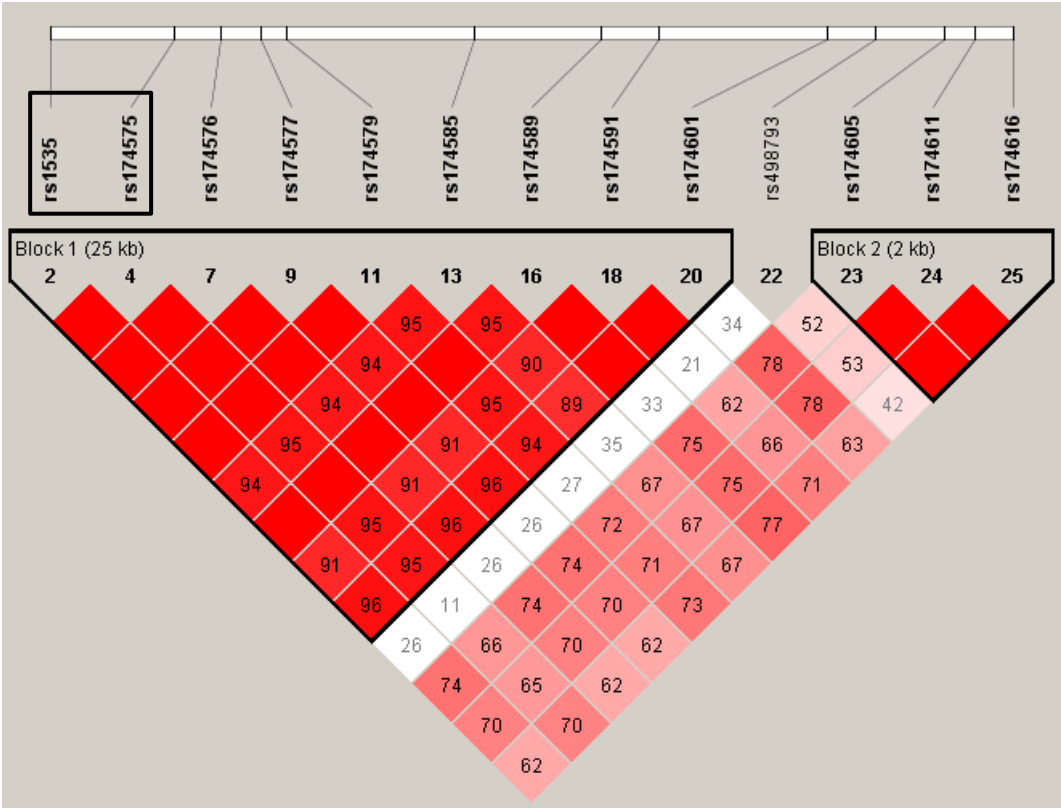

Supplement: Figure S2 — Linkage Disequilibrium map of variants in the FADS2 gene. The figure shows linkage disequilibria (D') for 26 variants of the FADS2 gene (part A) and for a subset of 13 common variants (minor allele frequency >0.2) (part B). The two polymorphisms, rs1535 and rs174575, are shown in boxes. Data source: HapMap release 21, CEPH database. (0.13 MB PDF) [file pone.0011570.s006.pdf]
